# Favorable revision-free survivorship of cemented arthroplasty following failed proximal femoral nail antirotation: a case series with a median follow-up of 10 years

**DOI:** 10.1186/s12891-022-05995-2

**Published:** 2022-11-29

**Authors:** Yi Li, Yaodong Zhang, Minji Yu, Tao Huang, Kunhong Li, Junxing Ye, Heng Huang, Weiguang Yu

**Affiliations:** 1 Department of Anesthesiology, Wuhan Fourth Hospital, No. 473, Hanzheng Street, Qiaokou District, Wuhan, 430030 China; 2 Department of Anesthesiology, The First Affiliated Hospital, Sun Yat-sen University, No. 58, Zhongshan 2nd Road, Yuexiu District, Guangzhou, 510080 China; 3 Department of Traditional Chinese Medicine, The First Affiliated Hospital, Sun Yat-sen University, No. 58, Zhongshan 2nd Road, Yuexiu District, Guangzhou, 510080 China; 4 Department of Orthopedics, Wuhan Third Hospital, Tongren Hospital of Wuhan University, No. 241, Pengliuyang Road, Wuchang District, Wuhan, 430060 China; 5 Department of Anesthesiology, Wuhan Fourth Hospital, No. 473, Hanzheng Street, Qiaokou District, Wuhan, China; 6 Department of Orthopedics, The Affiliated Hospital of Jiangnan University, No. 1000, Hefeng Road, Wuxi, 214000 Jiangsu China; 7 Department of Orthopedics, The First Affiliated Hospital, Sun Yat-sen University, No. 58, Zhongshan 2nd Road, Yuexiu District, Guangzhou, 510080 China

**Keywords:** Failure, Survivorship, Total hip arthroplasty, Complication, Cemented

## Abstract

**Background:**

Given the ever-increasing rate of failure related to proximal femoral nail antirotation (PFNA), it is expected that an increasing number of PFNA individuals will undergo conversion to total hip arthroplasty (THA). The long-term survivorship of conversion of the initial PFNA to cemented THA is still debated. The aim of this retrospective study was to assess the long-term revision-free survivorship of cemented THAs after initial failures of PFNA in geriatric individuals.

**Methods:**

Consecutive geriatric individuals who underwent secondary cemented THA after initial PFNA fixation from July 2005 to July 2018, were retrospectively identified from three medical centres. The primary outcome was revision-free survivorship estimated using the Kaplan–Meier method and Cox proportional hazards regression with revision for any reason as the endpoint; secondary outcomes were functional outcomes and key THA-related complications. Follow-ups occurred at 3 months, 6 months, 12 months and then every 12 months after conversion.

**Results:**

In total, 186 consecutive patients (186 hips) were available for study inclusion. The median follow-up was 120.7 months (60–180 months) in the cohort. Kaplan–Meier survivorship with revision for any reason as the end point showed that the 10-year revision-free survival rate was 0.852 (95% confidence interval [CI], 0.771–0.890). Good functional outcomes were seen, and the HHS decreased markedly over the 24th month to the final follow-up interval from 92.2 to 75.1 (each *p* < 0.05). The overall rate of key THA-related complications was 16.1% (30/186).

**Conclusion:**

Cemented THA executed following initial PFNA failure may yield satisfactory revision-free survival and, at least for the initial 10 years after conversion, good functional outcomes and a 16.1% complication rate of key THA-related complications, which supports the trend towards increased use of cemented THA.

## Background

Implant failure secondary to proximal femoral nail antirotation (PFNA) is a disabling complication of hip surgery [1–3]. Patients with failed PFNA are frequently accompanied by a noteworthy risk of death, increased cardia-cerebrovascular events, and reduced limb movement function and may experience conversion to total hip arthroplasty (THA) if there are no contraindications [4, 5]. Evidence-based best practice [6, 7] shows that THA is an expected solution to manage a failed PFNA, as it has reliable clinical outcomes and allows early rehabilitation and functional recovery, yet distrust remains as to a cemented or uncemented THA to apply in attempts to achieve superior clinical outcomes. With the burden of conversion to THA predicted to climb at a tremendous rate with the aging of the population, the survival of conversion of the initial PFNA to THA has been a growing concern [1, 8, 9]. This concern is further animated by the fact that the conversion to THA exposes patients to a leading challenge on the femur side, particularly when extensive bone loss and/or fractures occur [10]. Under such conditions, cemented THA may contribute significantly to enhancing hip stability and improving wear-resistant bearings [10, 11]. Patients experiencing cemented THA may achieve long-term prosthetic survival, as concerns related to prosthesis dislocations have been moderated with the application of larger-diameter heads and enhanced ligament patch-up methods [12, 13].

Previous studies [1, 14] note that the type of femoral prosthesis should be determined according to the lateral mass of the femur and the shape of the medullary cavity in salvage hip replacement. However, due to the hardening of the proximal femur and medullary cavity after removal of the intramedullary nail, it is difficult to anchor the bone cement into the bone bed of the femur and to form a more uniform cement sheath around the femoral stem, which can result in early fracture and failure of the bone cement sheath and increase the incidence of cement-related complications. In recent years, with the improvement of biological hip prostheses in surface coating treatment and stem design, the use rate of biological femoral stems in salvage hip replacement has gradually increased [15, 16]. However, for patients with severe osteoporosis or older than 70 years, cemented femoral stems may be more likely to be chosen by clinicians [1].

To date, there is a deficiency in data related to the long-term survival of cemented THA following initial PFNA failure. In addition, no consensus has been reached on the effect of conversion on the long-term survival of cemented THA. With this background, we retrospectively reviewed patients who experienced a conversion of primary PFNA to cemented THA to estimate the long-term revision-free survivorship of cemented THAs and to determine whether this conversion has improved long-term prosthesis survival in geriatric individuals. We hypothesize that conversion to cemented THA would be a satisfactory salvage procedure.

## Materials and methods

### Study population

Data on a consecutive cohort of individuals who experienced cemented THA after PFNA failure between July 2005 and July 2018 were retrospectively identified from three medical centres (Wuhan Fourth Hospital; The Affiliated Hospital of Jiangnan University; The First Affiliated Hospital, Sun Yat-sen University). The indication for conversion to cemented THA consisted of helical blade cutout and perforation, main nail breakage, and nonunion. The product details of PFNA and cemented THA are shown in Table 1. The surgical procedure and the postsurgical rehabilitation programme were in accordance with previous studies [1, 8, 9]. Comorbidities were assessed using the Charlson comorbidity index (CCI). Key exclusion criteria included unclear or deficient baseline data (i.e., unclear brand of prosthesis or cement, uncertain surgical indications), less than 5 years of follow-up, bilateral THA, conversion secondary to hip infection, osteomyelitis of the femur on the affected side, inability to ambulate independently after conversion to cemented THA and the reason for inability to ambulate independently being unrelated to conversion THA (i.e., medical problems, spinal disorders, ageing frailty), hip dysplasia (i.e., developmental dysplaisa of the hips), hemiplegia of the affected limb caused by stroke, sequelae of injury to nerve of the lower limb, poliomyelitis, spinal cord injury, Injury Severity Score > 16, cancer, an American Society of Anesthesiologists (ASA) physical status of 4 or 5, and psychiatric disorder.

**Table 1:** Product details of implants

	Stem	Cup	Cement type	PFNA
Cemented THA(*n* = 186)	Charnley^a^	Charnley^b^	Palacos-type cement	Synthes, Solothurn, Switzerland

^a^Zimmer, Warsaw, Indiana; ^b^a nonmodular head with a cemented full polyethylene acetabular socket. PFNA: proximal femoral nail anti-rotation

### Surgical procedures

The conversion to cemented THA was executed at every medical centre by 4–5 high-volume surgeons using the manufacturer’s instructions and recognized technical recommendations, as reported in previous studies [17, 18]. All surgeries were carried out through a transgluteal lateral approach. A third-generation cementing technique was used during the cemented THA procedure. The position of the bioresorbable distal cement restrictor was set beyond the distal screw hole from the PFNA extraction. Using a cement gun and digital pressure, we retrograde filled bone cement into the femoral marrow cavity to achieve clear penetration into the cancellous bone. The distal screw hole was bridged by the stem and distal cement mantle at an adequate length.

### Outcomes and variables

The assessment of baseline data was conducted by reviewing electronic medical records and follow-up reports. Primary surgeries and subsequent conversions were well documented. The primary outcome was revision-free survivorship estimated with the Kaplan–Meier method and Cox proportional hazards regression with revision for any reason as the endpoint. Revision was defined as the removal or exchange of at least 1 of the components [19]. The secondary outcomes comprised functional outcomes measured by Harris Hip Scores (HHS) and key THA-related complications (stem loosening, femoral fracture, and recurrent dislocation). Stem loosening was defined according to previous reports [20, 21]. Patients who were functioning were defined as having a functional score of 72 or greater. Follow-ups occurred at 3 months, 6 months, and 12 months and then every 12 months after conversion until the end of the study or death.

### Statistical analysis

Analyses were based on clinical data and patient follow-up during the study period. For categorical variables, the counts (N) and percentages (%) were expressed. For continuous variables, the mean ± SD (standard deviation) or median (range) was described, and comparisons of functional outcomes between the follow-up time points were performed with Student’s t-test. The type 1 error (alpha) was set at 0.05. Follow-up lasted from the date of conversion to cemented THA until failure or revision of cemented THA, death, or the end of the follow-up, whichever came first. Median follow-up was calculated with the reversed Kaplan-Meier method. Implant survival at 10 years was estimated using the Kaplan–Meier method with 95% confidence intervals (CIs). The survival rate was estimated using Cox proportional hazards regression with revision for any reason as the end point and with adjustment for age, sex, bone mineral density (BMD), time to revision, CCI at revision, and ASA physical status. Data quality was audited by two coauthors (WY and KL). Statistical analyses were performed with GraphPad Prism 8.4 (Inc., San Diego) or SAS 9.4 (SAS Institute, Cary, NC).

## Results

**Fig. 1:**
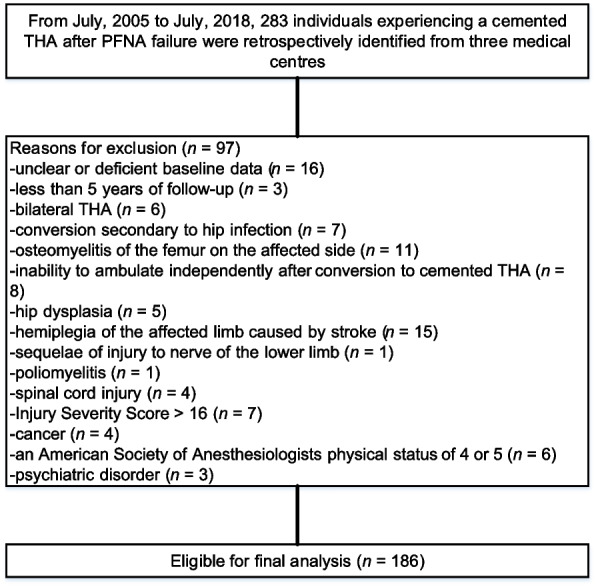
Flow diagram presenting the method for the identification of geriatric patients to assess the long-term survivorship of cemented THA after initial PFNA failure

From July 2005 to July 2018, a total of 283 consecutive patients were retrospectively reviewed. Among them, 97 patients were excluded based on our inclusion criteria, leaving 186 consecutive patients (186 hips) for inclusion in the study, as shown in Fig. 1. Table 2 shows a detailed breakdown of the baseline characteristics. Patient age at the time of index conversion was ≥70 years and < 80 years for 63.4% and ≥ 80 years for 36.6% in this cohort. A male predominance was seen in the overall sample (54.8%, 102/186), which was more prominent among individuals with high activity levels. Helical blade cutout and perforation were the most common indications for conversion to cemented THA, present in 88 of 186 patients, followed by nonunion in 71 of 186. Only 27 of 186 patients experienced conversion to cemented THA attributed to main nail breakage. The mean HHS prior to conversion was 65.0 ± 11.3.

**Table 2:** Patient characteristics at baseline

Variable	Cemented THA (*n* = 186)
Age (years), no.%	
70 ≤, < 80	118(63.4)
80 ≤	68(36.6)
Sex, no. %	
Male	102(54.8)
Female	84(45.2)
BMI (kg/m^2^)	
Median (range)	25.6 (17.9–32.7)
BMD (g/cm^3^)	3.81 ± 0.93
Side, no.%	
Left	96(51.6)
Right	90(48.4)
Time to conversion (months), no.%	
< 6	105(56.5)
≥ 6	81(43.5)
CCI at conversion, no. %	
Low	98(52.7)
Medium	64(34.4)
High	24(12.9)
Indications for conversion to a cemented THA, no. %	
Nonunion	71(38.2)
Helical blade cutout and perforation	88(47.3)
Main nail breakage	27(14.5)
ASA physical status, no.%	
1	49(26.3)
2	101(54.3)
3	36(19.4)
HHS prior to conversion	65.0 ± 11.3
Follow-up (months)	
Median (range)	120.7(60–180)

*THA* Total hip arthroplasty, *BMI* Body mass index, *BMD* Bone mineral density, *CCI* Charlson comorbidity index, *ASA* American Society of Anesthesiologists, *HHS* Harris hip scores

### Primary outcome

**Fig. 2:**
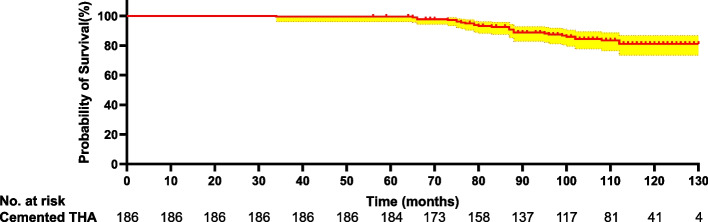
Kaplan–Meier survival curve with revision for any reason as the endpoint

The median follow-up was 120.7 months (60–180 months) in the cohort. Fig. 2 shows the overall survival curve for this cohort. Kaplan–Meier survivorship with revision for any reason as the end point showed that the 10-year revision-free survival rate was 0.852 (95% CI, 0.771–0.890). Of the 28 patients who underwent revision THA, 13 had stem loosening, 7 had recurrent dislocation, and 8 had a femoral fracture. The most frequent indication of revision was stem loosening.

### Secondary outcomes

**Fig. 3:**
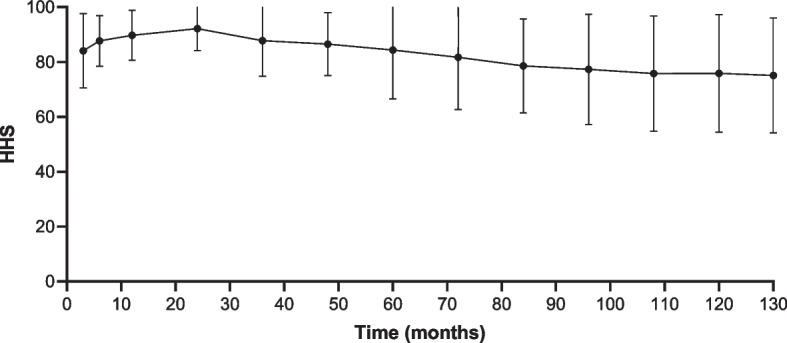
The variation trend of functional outcomes following conversion to cemented THA

**Fig. 4:**
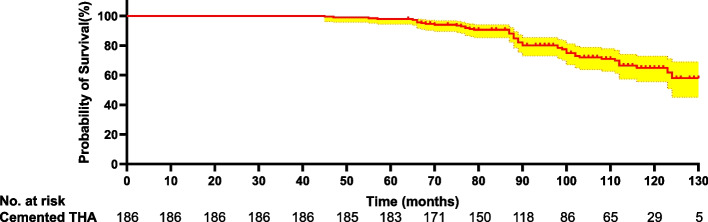
Kaplan-Meier survivorship with a functioning score of less than 72 as the endpoint

**Fig. 5:**
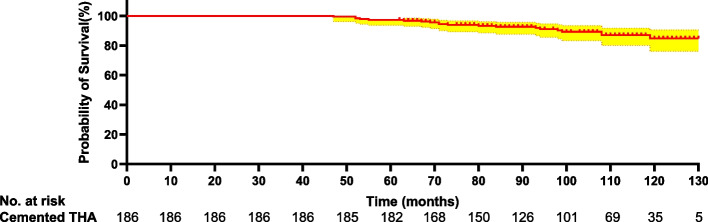
Kaplan–Meier survival curve with stem loosening as the endpoint

**Fig. 6:**
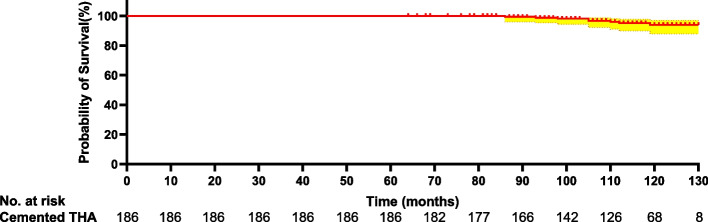
Kaplan–Meier survival curve with femoral fractures as the endpoint

Table 3 presents the long-term follow-up HHS. Fig. 3 provides a long-term trend of the HHS. Functional outcomes were assessed in 180 patients with at least 10 years of follow-up since the index conversion. The trend of deviation tended to increase from the 24th month onwards. The HHS in this cohort decreased markedly over the 24th month to the final follow-up interval from 92.2 to 75.1 (each *p* < 0.05). The HHS failed to illustrate a significant decline over the 108th month to the final follow-up interval from 76.3 to 75.1 (each *p* > 0.05). Fig. 4 shows the Kaplan-Meier survivorship with a functioning score of less than 72 as the endpoint. Table 4 provides an overview of the key THA-related complications related to conversion to cemented THAs in the present study. In this cohort, 28 (15.1%) patients underwent revision THA, 20 (10.7%) had stem loosening (Fig. 5), 7 (3.7%) had recurrent dislocation, and 8 (4.3%) suffered a femoral fracture (Fig. 6). Of the secondary 186 cemented THAs, one hundred fifty-eight (84.9%) were functioning at the final follow-up. Of the 158 cemented THAs in living patients, one hundred twenty-four (78.5%) functioned with the index components in position at least 10 years after conversion. Of the 186 patients, 30 patients had 35 THA-related complications. The overall complication rate was 16.1% (30/186).

**Table 3:** Functional outcomes of patients experiencing a conversion to cemented THA

Month(s) after conversion	Cemented THA^a^ (*n* = 186)
3	84.1 ± 9.6
6	87.7 ± 6.5
12	89.9 ± 6.3
24	92.2 ± 5.7
36	87.8 ± 9.2
48	86.5 ± 8.1
60	84.4 ± 12.6
72	81.7 ± 13.5
84	78.6 ± 12.1
96	77.3 ± 14.2
108	76.3 ± 15.3
120	75.9 ± 15.2
Final follow-up	75.1 ± 14.8

^a^Statistically significant values between postoperative functional outcomes and preoperative functional outcomes. *THA* Total hip arthroplasty

**Table 4:** Key THA-related complications in patients experiencing cemented THA.

Variable, no.%	Cemented THA (*n* = 186)
Revision	28(15.1)
Stem loosening	20(10.7)
Recurrent dislocation	7(3.7)
Femoral fracture	8(4.3)

*THA* Total hip arthroplasty

## Discussion

This retrospective multicenter study shows that conversion to cemented THA may yield satisfactory revision-free survival, at least for the initial 10 years after conversion, with improved functional outcomes and a 16.1% complication rate of key THA-related complications. These results confirm that conversion to cemented THA may contribute to enhancing the stability of the prosthesis and may remain a salvage procedure in geriatric individuals with failed PFNA.

Consistent with our findings, published data [22, 23] from the Nordic Arthroplasty Register Association (NARA) reported more than 90% of 10-year all-cemented THA survival in individuals aged 65–74 and more than 95% in individuals aged 75 or older. Similarly, a previous long-term study [20] of 62,305 cemented THAs in the Norwegian Arthroplasty Register showed that long-term implant survival was satisfactory for Charnley cemented THAs and improved significantly over time. An earlier study [24] of long-term survivorship of 2000 cemented THAs in 1689 individuals showed that the rate of survivorship with free of revision or removal of the implant for any reason as the end point was 86.5%. In addition, a brand-level study [22] of more than 360,000 patients experiencing primary cemented THAs showed that the survival of every brand implant was greater than 89% at 10 years and that Charnley cemented THA has a remarkable survival advantage over time, along with a low risk of revision. Although the number of comparable studies based on similar clinical settings is limited, several reports [25, 26] showed that mechanical enhancement with cemented THA may not have improved long-term survival. Although the use of cemented THA may be associated with low implant survival in geriatric individuals, their findings are constrained by a small sample size and a retrospective design and have not been confirmed by randomized controlled trials, and to date, there is, to our knowledge, no other evidence to support their conclusion.

Conversion to cemented THA is a complicated and laborious process, particularly in geriatric individuals who rely heavily on rapid recovery of early mobilization with full weight-bearing to combat a range of immobilization-related complications [1, 8, 27, 28]. However, cemented conversion may be associated with the femoral component loosening attributed to the combined axial loads and rotational moments [29]. Stem loosening can contribute to the failure of cemented THAs [30, 31], particularly in geriatric individuals with massive bone loss, although bone cement can, to some extent, enhance the component anchorage within the bone trabecula and results in improved functional outcomes with acceptable complications [32]. To date, limited literature [33, 34] has explored cement-related osteolysis, and there is no consensus on when it occurs, how long it lasts, and the extent and scope of its impact on the survival of the prosthesis. It is possible that cement augmentation for cemented THA is prone to promoting early mobilization in individuals with poor bone quality [35]. Nonetheless, evidence on the implant-bone stabilizing effect of cement augmentation is a controversial issue [36–38].

A growing body of evidence [1, 13, 22, 25, 39, 40] suggests that a cemented femur component can resist bone reconstruction or osteoporosis-induced stem loosening under the dual action of axial and rotational stresses, and bone cement can have a positive acceleration effect on bone cell apoptosis, which in turn leads to an increase in trabecular spacing, a decrease in bone mass, and the destruction of the bone reticular structure. Nevertheless, it remains uncertain whether bone reconstruction or osteoporosis-induced stem loosening continues after conversion to cemented THA or whether there is a positive correlation between bone reconstruction or osteoporosis-induced stem loosening and bone cement [41, 42], although previous reports [43, 44] have indicated that after conversion to cemented THAs, the improvement in joint stability is largely based on the reduction of osteoporosis-induced stem loosening. While not currently appreciated in some studies [8, 23], supplemental evidence will be indispensable in attempts to define whether osteoporosis-induced stem loosening is impeded in the long term with bone cement implantation. Additionally, patients experiencing conversion to cemented THA should deliberate on cement-related complications and subsequently the probability of conversion to cemented THA or balance the impending benefits of the cement components against the risk of rerevision.

Several drawbacks should be recognized in this study. First and most importantly, this is a retrospective observational study with inherent shortcomings. Comparison of implant survival can be influenced by strict inclusion criteria and surgical characteristics, leading to confounding results. Second, the conclusions may be limited to the multiformity of the definition and inclusion criteria, the relatively small sample size, and the lack of a control group. However, given the long-term follow-up data reported after conversion to cemented THAs, we believe that the current conclusions have some reference value for future conversion PFNA, although conclusions related to causality fail to be inferred. Third, the generalizability of the findings may be lacking since we only included geriatric individuals aged 70 years or older.

## Conclusions

This study demonstrated that cemented THA executed following initial PFNA failure may yield a satisfactory revision-free survivorship and, at least for the initial 10 years after conversion, with good functional outcomes and a 16.1% complication rate of key THA-related complications, which may support the trend towards increased use of cemented THA.

## Data Availability

All data generated or analysed during this study are not publicly available due to the protection of patient privacy but are available from the corresponding author upon reasonable request.

## Abbreviations

PFNA: Proximal femoral nail antirotation
THA: Total hip arthroplasty
CCI: Charlson comorbidity index
ASA: American Society of Anesthesiologists
HHS: Harris hip score
SD: Standard deviation
CIs: Confidence intervals
BMD: Bone mineral density

## References

[1] Yu W, Han X, Chen W, Mao S, Zhao M, Zhang X, Han G, Ye J, Chen M, Zhuang J (2020). Conversion from a failed proximal femoral nail anti-rotation to a cemented or uncemented total hip arthroplasty device: a retrospective review of 198 hips with previous intertrochanteric femur fractures. BMC Musculoskelet Disord.

[2] Zhang W, Xavier RPA, Decruz J, Chen YD, Park DH (2021). Risk factors for mechanical failure of intertrochanteric fractures after fixation with proximal femoral nail antirotation (PFNA II): a study in a southeast Asian population. Arch Orthop Trauma Surg.

[3] Goffin JM, Pankaj P, Simpson A, Seil R, Gerich TG (2013). Does bone compaction around the helical blade of a proximal femoral nail antirotation (PFNA) decrease the risk of cut-out? A SUBJECT-SPECIFIC COMPUTATIONAL STUDY. Bone & Joint Res.

[4] Pu JS, Liu L, Wang GL, Fang Y, Yang TF (2009). Results of the proximal femoral nail anti-rotation (PFNA) in elderly Chinese patients. Int Orthop.

[5] Hao YL, Zhang ZS, Zhou F, Ji HQ, Tian Y, Guo Y, Lv Y, Yang ZW, Hou GJ (2019). Risk factors for implant failure in reverse oblique and transverse intertrochanteric fractures treated with proximal femoral nail antirotation (PFNA). J Orthop Surg Res.

[6] Towle KM, Monnot AD (2016). An assessment of gender-Specific risk of implant revision after primary Total hip arthroplasty: a systematic review and Meta-analysis. J Arthroplast.

[7] Taylor JW, Frampton C, Rothwell AG (2018). Long-term survival of Total hip arthroplasty using implants from different manufacturers. J Arthroplast.

[8] Zeng X, Zhan K, Zhang L, Zeng D, Yu W, Zhang X, Zhao M (2017). Conversion to total hip arthroplasty after failed proximal femoral nail antirotations or dynamic hip screw fixations for stable intertrochanteric femur fractures: a retrospective study with a minimum follow-up of 3 years. BMC Musculoskelet Disord.

[9] Schultz BJ, Sicat C, Penev A, Schwarzkopf R, Egol KA. Conversion total hip arthroplasty for early failure following unstable intertrochanteric hip fracture: what can patients expect? Arch Orthop Trauma Surg. 2019:1–9. 10.1007/s00402-021-04215-1. 10.1007/s00402-021-04215-1 34657163

[10] Evans JT, Blom AW, Timperley AJ, Dieppe P, Wilson MJ, Sayers A, Whitehouse MR (2020). Factors associated with implant survival following total hip replacement surgery: a registry study of data from the National Joint Registry of England, Wales, Northern Ireland and the Isle of Man. PLoS Med.

[11] Davis CM, Berry DJ, Harmsen WS (2003). Cemented revision of failed uncemented femoral components of total hip arthroplasty. Journal of Bone and Joint Surgery-American Volume.

[12] Lachiewicz PE, Kelley SS, Soileau ES (2008). Survival of polished compared with precoated roughened cemented femoral components - a prospective, randomized study. Journal of Bone and Joint Surgery-American Volume..

[13] Makela KT, Eskelinen A, Pulkkinen P, Virolainen P, Paavolainen P, Remes V (2011). Cemented versus Cementless Total hip replacements in patients fifty-five years of age or older with rheumatoid arthritis. Journal of Bone and Joint Surgery-American Volume..

[14] Pallaver A, Zwicky L, Bolliger L, Bosebeck H, Manzoni I, Schadelin S, Ochsner PE, Clauss M (2018). Long-term results of revision total hip arthroplasty with a cemented femoral component. Arch Orthop Trauma Surg.

[15] Finch DJ, Martin BI, Franklin PD, Magder LS, Pellegrini VD, Investigators P (2020). Patient-reported outcomes following Total hip arthroplasty: a multicenter comparison based on surgical approaches. J Arthroplast.

[16] Siljander MP, Trousdale RT, Perry KI, Mabry TM, Berry DJ, Abdel MP (2021). Total hip arthroplasty in patients with Osteopetrosis. J Arthroplast.

[17] Hirose S, Otsuka H, Morishima T, Sato K (2012). Outcomes of Charnley total hip arthroplasty using improved cementing with so-called second- and third-generation techniques. J Orthop Sci.

[18] Sendtner E, Borowiak K, Schuster T, Woerner M, Grifka J, Renkawitz T (2011). Tackling the learning curve: comparison between the anterior, minimally invasive (Micro-hip(a (R))) and the lateral, transgluteal (Bauer) approach for primary total hip replacement. Arch Orthop Trauma Surg.

[19] Cnudde P, Bulow E, Nemes S, Tyson Y, Mohaddes M, Rolfson O (2019). Association between patient survival following reoperation after total hip replacement and the reason for reoperation: an analysis of 9,926 patients in the Swedish hip arthroplasty register. Acta Orthop.

[20] Espehaug B, Furnes O, Engesaeter LB, Havelin LI (2009). 18 years of results with cemented primary hip prostheses in the Norwegian arthroplasty register concerns about some newer implants. Acta Orthop.

[21] Harris WH, McCarthy JC, O'Neill DA (1982). Femoral component loosening using contemporary techniques of femoral cement fixation. J Bone Joint Surg Am.

[22] Junnila M, Laaksonen I, Eskelinen A, Pulkkinen P, Havelin LI, Furnes O, Fenstad AM, Pedersen AB, Overgaard S, Karrholm J, Garellick G, Malchau H, Makela KT (2016). Implant survival of the most common cemented total hip devices from the Nordic arthroplasty register association database. Acta Orthop.

[23] Makela KT, Matilainen M, Pulkkinen P, Fenstad AM, Havelin L, Engesaeter L, Furnes O, Pedersen AB, Overgaard S, Karrholm J, Malchau H, Garellick G, Ranstam J, Eskelinen A (2014). Failure rate of cemented and uncemented total hip replacements: register study of combined Nordic database of four nations. Bmj-British Medical Journal.

[24] Berry DJ, Harmsen WS, Cabanela ME, Morrey BF (2002). Twenty-five-year survivorship of two thousand consecutive primary Charnley total hip replacements: factors affecting survivorship of acetabular and femoral components. J Bone Joint Surg Am.

[25] Gwynne-Jones DP, Gray AR (2020). Cemented or uncemented acetabular fixation in combination with the Exeter universal cemented stem LONG-TERM SURVIVAL TO 18 YEARS. Bone & Joint Journal.

[26] Meding JB, Galley MR, Ritter MA (2010). High survival of Uncemented proximally porous-coated titanium alloy femoral stems in osteoporotic bone. Clin Orthop Relat Res.

[27] Mathur HH, Shah HS, Vishwanathan K (2022). Functional outcome of conversion total hip arthroplasty (CTHA) using uncemented distally loading femoral stem for failed fixation of proximal femoral nail - a case series. J Orthop.

[28] Mueller F, Galler M, Zellner M, Baeuml C, Fuechtmeier B (2017). Total hip arthroplasty after failed osteosynthesis of proximal femoral fractures: revision and mortality of 80 patients. J Orthop Surg.

[29] Watts CD, Abdel MP, Hanssen AD, Pagnano MW (2016). Anatomic hip center decreases aseptic loosening rates after Total hip arthroplasty with cement in patients with Crowe type-II dysplasia a concise follow-up report at a mean of thirty-six years. Journal of Bone and Joint Surgery-American Volume..

[30] Lombardi AV, Mallory TH, Vaughn BK, Drouillard P (1989). Aseptic loosening in total hip arthroplasty secondary to osteolysis induced by wear debris from titanium-alloy modular femoral heads. J Bone Joint Surg Am.

[31] Acklin YP, Berli BJ, Frick W, Elke R, Morscher EW (2001). Nine-year results of Müller cemented titanium straight stems in total hip replacement. Arch Orthop Trauma Surg.

[32] Kovac S, Trebse R, Milosev I, Pavlovcic V, Pisot V (2006). Long-term survival of a cemented titanium-aluminium-vanadium alloy straight-stem femoral component. Journal of Bone and Joint Surgery-British Volume.

[33] Schuh A, Thomas P, Holzwarth U, Zeiler G (2004). Bilateral localized osteolysis after cemented total hip replacement. Orthopade..

[34] Scholl E, Eggli S, Ganz R (2000). Osteolysis in cemented titanium alloy hip prosthesis. J Arthroplast.

[35] Rogers BA, Kuchinad R, Garbedian S, Backstein D, Gross AE, Safir OA (2015). Cement augmentation of the acetabulum for revision Total hip arthroplasty for infection. J Arthroplast.

[36] Tsai SW, Chen CF, Wu PK, Chen CM, Chen WM (2018). Cement augmentation in the proximal femur to prevent stem subsidence in revision hip arthroplasty with Paprosky type II/IIIa defects. J Chin Med Assoc.

[37] Sermon A, Hofmann-Fliri L, Richards RG, Flamaing J, Windolf M (2014). Cement augmentation of hip implants in osteoporotic bone: how much cement is needed and where should it go?. J Orthop Res.

[38] Gisep A, Curtis R, Flutsch S, Suhm N (2006). Augmentation of osteoporotic bone: effect of pulsed jet-lavage on injection forces, cement distribution, and push-out strength of implants. Journal of Biomedical Materials Research Part B-Applied Biomaterials.

[39] Keeling P, Howell JR, Kassam AM, Sathu A, Timperley AJ, Hubble MJW, Wilson MJ, Whitehouse SL (2020). Long-term survival of the cemented Exeter universal stem in patients 50 years and younger: an update on 130 hips. J Arthroplast.

[40] Schulz KS, Nielsen C, Stover SM, Kass PH (2000). Comparison of the fit and geometry of reconstruction of femoral components of four cemented canine total hip replacement implants. Am J Vet Res.

[41] Schmalzried TP, Zahiri CA, Woolson ST (2000). The significance of stem-cement loosening of grit-blasted femoral components. Orthopedics..

[42] Harris B, Owen JR, Wayne JS, Jiranek WA (2010). Does femoral component loosening predispose to femoral fracture? An in vitro comparison of cemented hips. Clin Orthop Relat Res.

[43] Ota J, Cook JL, Lewis DD, Tomlinson JL, Fox DB, Cook CR, Schultz LG, Brumitt J (2005). Short-term aseptic loosening of the femoral component in canine total hip replacement: effects of cementing technique on cement mantle grade. Vet Surg.

[44] Mann KA, Damron LA, Miller MA, Race A, Clarke MT, Cleary RJ (2007). Stem-cement porosity may explain early loosening of cemented femoral hip components: experimental-computational in vitro study. J Orthop Res.

